# Comparison of long-term outcomes between Lynch sydrome and sporadic colorectal cancer: a propensity score matching analysis

**DOI:** 10.1186/s12885-020-07771-8

**Published:** 2021-01-09

**Authors:** Yun Xu, Cong Li, Charlie Zhi-Lin Zheng, Yu-Qin Zhang, Tian-An Guo, Fang-Qi Liu, Ye Xu

**Affiliations:** 1 Department of Colorectal Surgery, Fudan University Shanghai Cancer Center, No. 270 Dong-An Road, Shanghai, 200032 China; 2 Department of Oncology, Shanghai Medical College, Fudan University, Shanghai, 200032 China; 3 Mechanical and Aerospace Engineering, University of California, 7400 Boelter Hall, Los Angeles, CA 90095 USA

**Keywords:** Colorectal cancer, Lynch syndrome, Mismatch repair, Survival, Chemotherapy

## Abstract

**Background:**

Lynch syndrome (LS) is the most common hereditary colorectal cancer (CRC) syndrome. Comparison of prognosis between LS and sporadic CRC (SCRC) were rare, with conflicting results. This study aimed to compare the long-term outcomes between patients with LS and SCRC.

**Methods:**

Between June 2008 and September 2018, a total of 47 patients were diagnosed with LS by genetic testing at Fudan University Shanghai Cancer Center. A 1:2 propensity score matching was performed to obtain homogeneous cohorts from SCRC group. Thereafter, 94 SCRC patients were enrolled as control group. All of enrolled patients received curative surgeries and standardized postoperative monitoring. The long-term survival rates between the two groups were compared, and the prognostic factors were also analyzed.

**Results:**

The 5-year overall survival rate of LS group was 97.6%, which was significantly higher than of 82.6% for SCRC group (χ^2^ = 4.745, *p* = 0.029). The 5-year recurrence free survival rate showed no significant differences between the two groups (78.0% for LS group vs. 70.6% for SCRC patients; χ^2^ = 1.260, *p* = 0.262). The 5-year tumor free survival rates in LS group was 62.1% for LS patients, which were significantly lower than of 70.6% for SCRC group (χ^2^ = 4.258, *p* = 0.039). Subgroup analysis of recurrent patients show that the LS group had longer overall survival than the SCRC group after combined chemotherapy. By multivariate analysis, we found that tumor recurrence of primary CRC [Risk ratio (95% (confidence interval): 48.917(9.866–242.539); *p* < 0.001] and late TNM staging [Risk ratio (95% (confidence interval): 2.968(1.478–5.964); *p* = 0.002] were independent risk factors for OS.

**Conclusion:**

LS patients have better long-term survival prognosis than SCRC patients, even though the two groups have statistically comparable recurrence free survival. Combined chemotherapy is an effective treatment for LS patients who developed primary CRC recurrence. Standardized postoperative monitoring for LS patients may enable detection of metachronous tumors at earlier stages, which was a guarantee of a favorable prognosis despite lower tumor free survival.

## Background

Colorectal cancer (CRC) is one of the most frequently diagnosed malignancies worldwide and the second leading cause of cancer-related death globally [1]. CRC has been recognized as a heterogeneous disease based on different molecular mechanisms, therefore presenting heterogeneous outcomes and drug responses [2–4]. Lynch syndrome (LS) is the most common hereditary CRC syndrome. It results from heterozygous pathogenic germline variants in the mismatch repair (MMR) genes (path_*MLH1*, path_*MSH2*, path_*MSH6*, and path_*PMS2*), accounting for approximately 3–5% of all cases of CRC [5, 6]. In addition to higher risk for CRC, LS presents significantly higher risks for cancers in organs including the endometrium, ovaries, stomach, small bowel, bile duct, pancreas and upper urinary tract [5, 6]. The consequent tumors present the phenotypes of MMR protein deficiency (dMMR) under immunohistochemistry (IHC) and microsatellite instability (MSI).

During the last decade, multi-gene cancer panel tests on suspected LS patients have allowed the identification of LS at an increasing rate. Correspondingly, the introduction of tailored policies of management and treatment, different from those for sporadic colorectal cancer (SCRC), has largely contributed to the long-term prognosis of LS patients and their affected relatives.

In theory, CRC with dMMR should have better prognosis and therapeutic responses because the MMR pathway is involved in triggering cell death after chemotherapy-induced DNA damage [4, 7–10]. Although analysis of LS patients’ survival prognoses has been reported by several western studies [7–17], few studies compared the long-term prognoses of LS with that of SCRC, and those that did had conflicting results. In addition, almost all previous findings on LS were derived from Western medical centers, and data from Asian populations remain lacking.

The aim of this study is to compare the long-term survival outcome of LS associated CRC patients with that of SCRC patients. A propensity score matching (PSM) analysis was used to balance the baseline of the two groups. Afterwards, overall survival (OS), recurrence free survival (RFS), and tumor free survival (TFS) were compared, and prognostic factors associated with survival were also analyzed. To our knowledge, the current article is the first report on survival of LS from one of the largest colorectal surgery centers in China, and thus may better inform the comprehensive understanding of LS and clarify the differences between LS and SCRC.

## Methods

### Patients

Between June 2008 and September 2018, a total of 22,833 consecutive CRC patients underwent curative surgeries, depending on the location of tumors, at the Fudan University Shanghai Cancer Centre.

As in our clinical routine, patients who met at least one of the following two criteria were defined as suspected LS patients: 1) dMMR positive under IHC; 2) family history fulfills clinical criteria, including the Amsterdam criteria [18] and Bethesda guidelines [19]. Multi-gene panel testing that included 139 genes was recommended for suspected LS patients and some of their affected relatives. All patients gave informed consent for genetic analyses. Germline variants were defined as variants carried by both patients and their respective family members, for whom genetic counseling was recommended. Of the 252 suspected LS patients that underwent genetic testing, 47 were identified as carrying a pathogenic variant (PV) in MMR genes. They were diagnosed with LS and classified as LS group. SCRC was defined as patients with neither family history nor dMMR phenotype. Based on the baseline of LS group, we matched 94 SCRC patients (1:2) as SCRC group by propensity score matching.

### Immunohistochemistry

IHC were performed for all patients who underwent curative surgeries. MMR deficiency was determined according to the absence of protein expression for any one of several genes including *hMLH1*, *hMSH2*, *hMSH6*, and *hPMS2*. IHC was performed using the fully automated BenchMark ULTRA platform (Ventana Medical Systems, Inc., Tucson, AZ, United States). Normal tissues adjacent to the tumor or lymphocytes in the stroma served as internal positive controls. Each result was confirmed by at least two experienced pathologists.

### Mutation screening of KRAS, NRAS and BRAF

Mutation screening of *KRAS*, *NRAS* and *BRAF* were performed for all patients who underwent curative surgeries. The methods of mutation detection in *KRAS, NRAS* and *BRAF* were the same as our previous report [20]. All results were confirmed according to the criterion suggested by the manufacturer.

### Next-generation sequencing

Peripheral blood (10 mL) was collected, stored in ethylenediaminetetraacetic acid tubes, and allowed to stand at 25 °C for 2 h. The supernatant was transferred to a 15-mL centrifuge tube and then centrifuged for 10 min at 2200 g at 4 °C. Thereafter, the intermediate white blood cells were transferred to a 1.5-ml centrifuge tube. The DNA was recovered using the MagPure FFPE DNA LQ Kit (Magen). NGS was conducted on the germline DNA as a standard genetic testing for germline analysis.

Sequence data were mapped to the reference human genome (hg19) using BWA aligner 0.7.10. Local alignment optimization was performed using GATK 3.2. Germline SNVs were identified using Varscan with default parameters. Germline indels were identified using Varscan and GATK. Pathogenic variants were determined by a clinical molecular geneticist according to the guidelines of the American College of Medical Genetics [21]. The ClinVar (https://www.ncbi.nlm.nih.gov/clinvar/) was used during manual curation for final confirmation of the results. The InSIGHT database (https://www.insight-group.org/variants/databases/) was used for the pathogenicity classification of the MMR genes.

### Clinical data acquisition and follow-up evaluation

For the 141 enrolled patients, the baseline information on tumor characteristics, pathological results, and treatment were retrospectively obtained from medical charts. Follow-ups conducted for LS patients were according to clinical practice guidelines [22], and regular follow-ups were performed for SCRC patients. For all patients, contrast-enhanced abdominal CT/MRI and carcinoembryonic antigen (CEA) test were performed every 2–3 months within the first 1 year after surgery and every 4–6 months thereafter, to monitor tumor recurrence. Chest radiography was taken annually to detect lung metastasis. Colonoscopy was performed 1 year after surgery for all patients, then annually for LS patients and every 2–3 years for SCRC patients. For female LS patients, routine follow-ups also included annual gastroscopy, breast ultrasonography and gynecological examination. During the follow-up evaluations, any occurrence of tumor recurrence of primary CRC, metachronous CRC, and extra-colonic cancer was recorded.

Tumor recurrence was defined as any recurrent tumor at the anastomotic site, invasion of adjacent tissues, lymph node metastasis, or distant metastasis that developed within 5 years after surgery. Synchronous tumors were defined as two colorectal tumors that were discovered simultaneously or within 6 months of each other, and the metachronous colorectal tumors were discovered more than 6 months apart [23]. For patients with synchronous tumors, the tumor with the higher stage was documented as the primary tumor [17]. Extra-colonic cancers were defined as primary cancers within the disease spectrum of LS and presenting dMMR under IHC. This study was censored on July 31, 2020. The mean follow-up period was (80.3 ± 41.2) months for SCRC patients and (82.0 ± 57.2) months for LS patients, no significant difference was observed between the two groups in follow-up period (χ^2^= 0.238, *p* = 0.626).

### Analysis of outcome

The outcomes of this analysis were oncologic outcomes including OS, RFS, and TFS. OS time was defined as the period between the date of surgery to the date of death or last follow-up. RFS time was defined as the period between the date of surgery and the date of tumor recurrence or last follow-up. TFS time was defined as the period between the date of surgery and the date of tumor recurrence, metachronous CRC, extra-colonic cancer or last follow-up. Treatment options involving repeat resection, chemotherapy, radiotherapy, immunotherapy, conservative treatment for these events were formulated based on the recommendations of our multidisciplinary team. The primary endpoint was the 5-year OS rate; the secondary endpoints were 5-year TFS and RFS rates.

### Statistical analysis

All analyses were performed using the R software package (version 3.0.2; R Foundation for Statistical Computing, Vienna, Austria) and SPSS (statistical software (version 20.0; Chicago, Ill).

Propensity score matching analysis was performed using the R software package. We used propensity score matching to balance the assignment of the included patients. Patients were matched using the following baseline characteristics as covariates: age, gender, CEA, tumor location, tumor size, pathologic result (classification, differentiation grade, cancerous node, vascular invasion, perineural invasion), TNM stage, KRAS (wild type vs. variant type), NRAS (wild type vs. variant type), adjuvant chemotherapy (received or not) (Table 1). Each variable was multiplied by a coefficient that was calculated using logistic regression analysis, and the sum of these values was taken as the propensity score for individual patients. For matching, complex LS and SCRC pairs with an equivalent propensity score were selected by a 1: 2 matching. Thereafter, we matched 47 LS patients with 94 SCRC patients using the nearest neighbor method (caliber = 0.02).

**Table 1:** Demographic and clinical characteristics of 141 colorectal cancer patients

Variables	LS group (*N* = 47)	SCRC group (*N* = 94)	χ^2^ value	*p* value
Age (years)			0.070	0.792
< 50	35(74.5%)	68(72.3%)		
≥50	12(2.5.5%)	26(27.7)		
Gender			0.058	0.810
Male	26(55.3%)	54(57.4%)		
Female	21(44.7%)	40(42.6%)		
CEA (ng/ml)			0.104	0.747
≥5.2	7(14.9%)	16(17.0%)		
< 5.2	40(85.1%)	78(83.0%)		
Location			3.177	0.365
Right colon	18(38.3%)	40(42.6%)		
Left colon	20(42.6%)	41(43.6%)		
Rectal	5(10.6%)	11(11.7%)		
Multiple	4(8.5%)	2(2.1%)		
Tumor size ^a^ (cm)	5.17±2.61	5.21±2.79	0.106	0.754
Pathological classification			0.474	0.789
Adenocarcinoma	34(72.3%)	71(75.5%)		
Partial mucinous	5(10.7%)	11(11.7%)		
Mucinous adenocarcinoma	8(17.0%)	12(12.8%)		
Differentiation grade			0.259	0.878
Well	1(2.1%)	1(1.1%)		
Moderately	28(59.6%)	56(59.5%)		
Poorly	18(38.3%)	37(39.4%)		
Cancerous node			0.534	0.465
Occurrence	2(4.3%)	7(7.4%)		
Absence	45(95.7%)	87(92.6%)		
Vascular invasion			0.534	0.461
Occurrence	8(17.0%)	21(22.3%)		
Absence	39(83.0%)	73(77.7%)		
Perineural invasion			0.252	0.616
Occurrence	6(12.8%)	15(16.0%)		
Absence	41(87.2%)	79(84.0%)		
T stage			0.130	0.937
T1	7(14.9%)	12(12.8%)		
T2	8(17.0%)	17(18.1%)		
T3	32(68.1%)	65(69.1%)		
N stage			1.840	0.399
N0	34(72.3%)	57(60.6%)		
N1	9(19.1%)	23(24.5%)		
N2	4(8.6%)	14(14.9%)		
Metastasis			0.265	0.607
Occurrence	2(4.3%)	6(6.4%)		
Absence	45(95.7%)	88(93.6%)		
TNM stage			1.200	0.753
I	13(27.7%)	27(28.7%)		
II	17(36.2%)	30(31.9%)		
III	15(31.8%)	31(33.0%)		
IV	2(4.3%)	6(6.4%)		
*KRAS* mutant	21(44.6%)	49 (52.1%)	0.695	0.404
*NRAS* mutant	2(4.3%)	3 (3.2%)	0.104	0.747
Adjuvant chemotherapy			1.420	0.233
Received	26(55.3%)	42(44.7%)		

*CEA* Carcinoembryonic antigen, *LS* Lynch syndrome, *SCRC* Sporadic colorectal cancer

We assessed the balance of all baseline covariates in Table 1 between the two groups after propensity score matching. Continuous variables were compared using the Student t test between the two groups. Categorical variables were compared using the Chi square test or Fisher’s exact test. OS, TFS, and RFS curves were evaluated using Kaplan-Meier curves and compared using the log-rank test. Variables with *p*-values less than 0.05 in the univariate analysis were entered into a Cox proportional hazards model for multivariate analysis. For all statistical tests, two-tailed p-values less than 0.05 were considered statistically significant.

## Results

### Molecular characteristics

In the LS group, PVs of *MLH1* were identified in 17 (36.2%) probands and those of *MSH2*, *MSH6*, and *PMS2* were identified in 18 (38.3%), 10 (21.3%), and 2 (4.2%) probands, respectively. Variants from LS patients and frequency of each variant in the Asian population are summarized in Table 2. In patients identified with PVs in MMR genes, the results of IHC MMR staining were consistent with those of gene detection.

**Table 2:** Variants in the LS patients and frequency of each variant in Asian population

Gene	Variants (HGVS)	Clinical Significance	Frequency
*MLH1*	*NM*_*001167618.2(MLH1):c.-33del(p.Ile231fs)*	Pathogenic	No data
*MLH1*	*NM_000249.3(MLH1):c.1976G>C (p.Arg659Pro)*	Pathogenic	0.00002
*MLH1*	*NM_000249.3(MLH1):c.883A>G (p.Ser295Gly)*	Pathogenic	No data
*MLH1*	*NM_000249.4(MLH1):c.244A>G (p.Thr82Ala)*	Likely pathogenic	0.00000
*MLH1*	*NM_000249.3(MLH1):c.979C>T (p.Gln327Ter)*	Pathogenic	0.0000
*MLH1*	*NM_000249.3(MLH1):c.1489_1490insCG(p.Arg497fs)*	Pathogenic	No data
*MLH1*	*NM_000249.3(MLH1):c.199G>C (p.Gly67Arg)*	Pathogenic	0.0000
*MLH1*	*NM_000249.3(MLH1):c.2101C>A (p.Gln701Lys)*	Likely pathogenic	0.00249
*MLH1*	*NM_001167618.2(MLH1):c.-131_-130GA(p.Glu199fs)*	Pathogenic​	No data
*MLH1*	*NM_000249.3(MLH1):c.116+1G>A(5 prime UTR)*	Likely pathogenic​	No data
*MLH1*	*NM_000249.3(MLH1):c.1990-2A>G*	Pathogenic​	No data
*MLH1*	*NM_000249.3(MLH1):c.453+1G>T*	Likely pathogenic​	No data
*MLH1*	*NM_000249.3(MLH1):c.250A>G (p.Lys84Glu)*	Likely pathogenic​	No data
*MSH2*	*NM_000251.3(MSH2):c.1165C>T (p.Arg389Ter)*	Pathogenic​	No data
*MSH2*	*NM_000251.2(MSH2):c.244A>T (p.Lys82Ter)*	Pathogenic​	No data
*MSH2*	*NM_000251.2(MSH2):c.877A>G (p.Thr293Ala)*	Likely pathogenic​	0.0000
*MSH2*	*NM_000251.3(MSH2):c.2038C>T (p.Arg680Ter)*	Pathogenic​	0.0000
*MSH2*	*NM_000251.2(MSH2):c.1528C>T (p.Gln510Ter)*	Pathogenic​	No data
*MSH2*	*NM_000251.2(MSH2):c.1963G>A (p.Val655Ile)*	Likely pathogenic​	0.00012
*MSH2*	*NM_000251.2(MSH2):c.859G>T (p.Gly287Ter)*	Pathogenic​	No data
*MSH2*	*NM_000251.2(MSH2):c.1077A>T (p.Arg359Ser)*	Pathogenic	No data
*MSH2*	*NM_000251.3(MSH2):c.1710 T>G (p.Tyr570Ter)*	Pathogenic	No data
*MSH2*	*NM_000251.2(MSH2):c.352dup (p.Tyr118fs)*	Pathogenic	No data
*MSH2*	*NM_000251.2(MSH2):c.1009C>T (p.Gln337Ter)*	Pathogenic	No data
*MSH2*	*NM_000251.3(MSH2):c.2131C>T (p.Arg711Ter)*	Pathogenic	0.0000
*MSH2*	*NM_000251.2(MSH2):c.1042C>T (p.Gln348Ter)*	Pathogenic	No data
*MSH2*	*NM_000251.2(MSH2):c.2021G>A (p.Gly674Asp)*	Likely pathogenic​	No data
*MSH6*	*NM_000179.2(MSH6):c.3252dup (p.Thr1085fs)*	Pathogenic	No data
*MSH6*	*NM_000179.2(MSH6):c.718C>T (p.Arg240Ter)*	Pathogenic	0.0000
*MSH6*	*NM_000179.2(MSH6):c.2294dup (p.Cys765fs)*	Pathogenic	No data
*MSH6*	*NM_000179.2(MSH6):c.3515G>C (p.Arg1172Thr)*	Pathogenic	No data
*MSH6*	NM_000179.3(MSH6):c.3202C>T (p.Arg1068*)	Pathogenic​	No data
*MSH6*	*NM_000179.2(MSH6):c.652A>T (p.Lys218Ter)*	Pathogenic	0.00000
*MSH6*	*NM_000251.2(MSH2):c.518 T>C (p.Leu173Pro)*	Likely pathogenic​	No data
*PMS2*	*large intragenic in EXON9*	Pathogenic	No data
*PMS2*	*NM_000535.7:c.2 T>G(p.Met1Arg)*	Pathogenic​	0.000

*LS* Lynch syndrome

### Patient groups

After propensity score 1:2 matching analysis, 94 SCRC patients were enrolled as control group. The baseline characteristics of these two groups were compared and summarized in the Table 1, and no significant difference was found between the two groups in any variables. In the LS group, synchronous CRC were observed in four (4/47, 8.5%) patients. However, the occurrence of synchronous tumors is a major clinical feature intrinsic to LS and is rarely observed in SCRC patients. Thus, this variable did not serve as a baseline for comparison.

### Recurrence of primary CRC and recurrence free survival

**Fig. 1:**
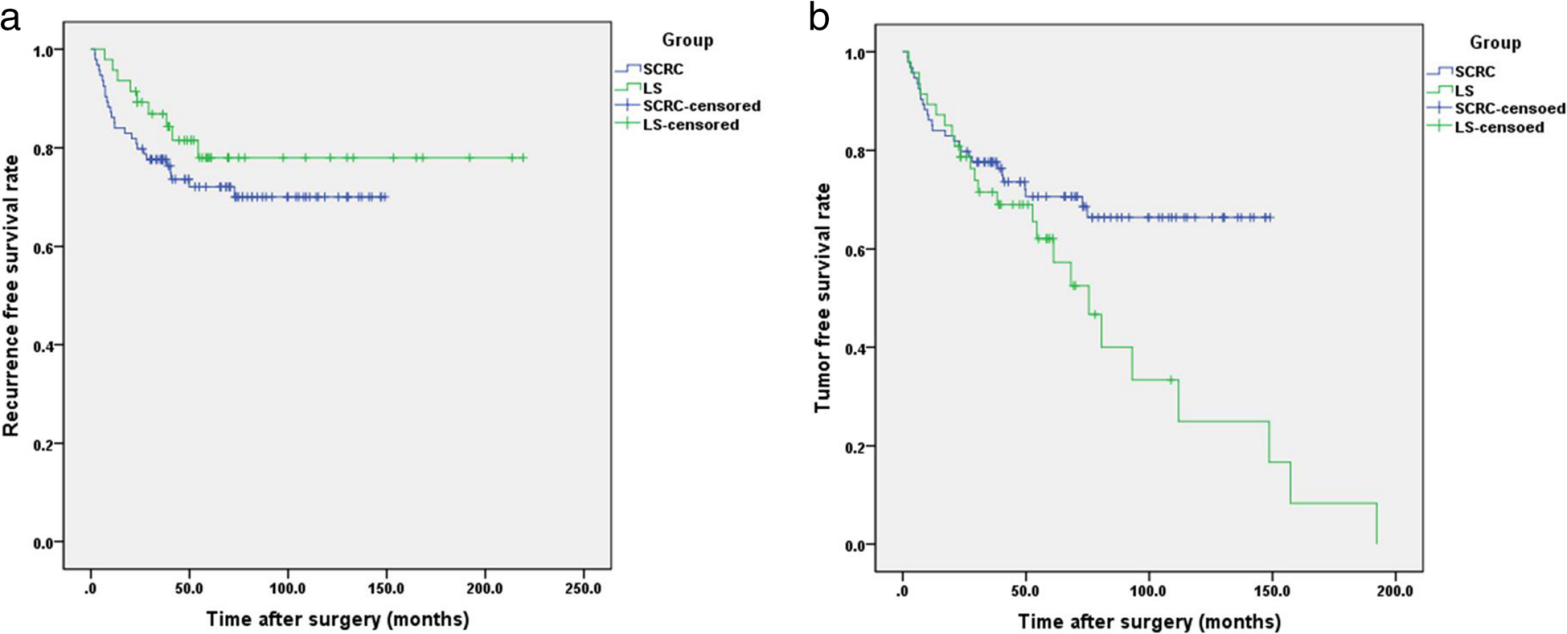
Recurrence free survival curves (**a**) and tumor free survival curves (**b**) for patients in LS group and SCRC group. Survivals were evaluated using Kaplan–Meier curves and compared with the log-rank test

During the follow up, a total of 26 (27.7%) SCRC patients developed tumor recurrence including 14 (14.9%) of liver metastases, 4 (4.3%) abdominal lymph node metastasis, 3 (3.2%) of lung metastases, 3 (3.2%) of invasion of adjacent tissues, and 2 (2.1%) of extensive metastasis. In LS group, 9 (19.1%) patients developed tumor recurrence including 4 (8.5%) of liver metastases, 2 of (4.3%) of invasion of adjacent tissues, and 2 (4.3%) of abdominal lymph node metastasis. No significant differences were observed between the two groups (χ^2^ = 1.216, *p* = 0.270). The average RFS period was (110.8 ± 61.8) months [95% confidence interval (CI): 98.3–123.3] for SCRC group and (177.3±85.9) months (95% CI: 152.8–201.9) for LS group. The 1-, 3-, and 5-year RFS rates for the SCRC group were 85.1, 77.6, and 70.6%, respectively, whereas those for the LS group were 95.7, 86.9, and 78.0%, respectively. No significant differences were observed between the two groups in RFS (χ^2^ = 1.260, *p* = 0.262, Fig. 1a).

All patients who developed tumor recurrence in both groups received 5-fluorouracil based chemotherapy (XELOX or mFOLFOX6). In LS group, all patients received targeted agents, including cetuximab for 3 patients with wild-type KRAS and bevacizumab for 6 patients with variant-type KRAS. In SCRC group, cetuximab combined with chemotherapy was performed for 4 patients with wild-type KRAS and bevacizumab for 7 patients with variant-type KRAS.

### Metachronous tumor and tumor free survival

During the follow-up period, 34.0% (16/47) of the patients in the LS group developed metachronous CRC, with average period of (28.78 ± 29.14) months between the occurrence of primary and metachronous CRC. In addition, 11 patients developed 15 cases of primary extra-colonic cancer, including 5 cases of endometrial cancer, 5 cases of gastric cancer, 2 cases of small intestinal cancer, and 1 case each of ovarian, breast, and cutaneous cancer. Therefore, the average TFS period was (82.4±78.7) months (95% CI: 59.9–104.9) for LS group and (107.7±63.1) months (95% CI: 94.9–120.5) for SCRC group. In LS group, the 1-, 3-, and 5-year TFS rates were 89.4, 71.5, and 62.1% respectively, which were significantly lower than those in SCRC group (85.1, 77.6, and 70.6%, respectively; χ^2^ = 4.258, *p* = 0.039) (Fig. 1b).

All patients with metachronous cancers received radical resection. Of the 16 patients who developed metachronous CRC, 14 patients underwent extended resection, including 9 cases of subtotal colectomy, 4 of extended left hemicolectomy, 1 of total colectomy, and 1 of extended right hemicolectomy; the other 2 patients underwent standard radical resection. 5-fluorouracil based adjuvant chemotherapy was performed for 2 patients in stage II and 5 patients in stage III.

### Overall survival

During the follow-up period, 4 (8.5%) LS patients, including 2 *MLH1* variants carriers and 2 *MSH2* variants carrier, died of tumor recurrence. No significant differences in OS were found among the four genotypes (χ^2^ = 3.803 *p* = 0.430). In SCRC group, 24 (25.5%) SCRC patients died of tumor recurrence.

**Fig. 2:**
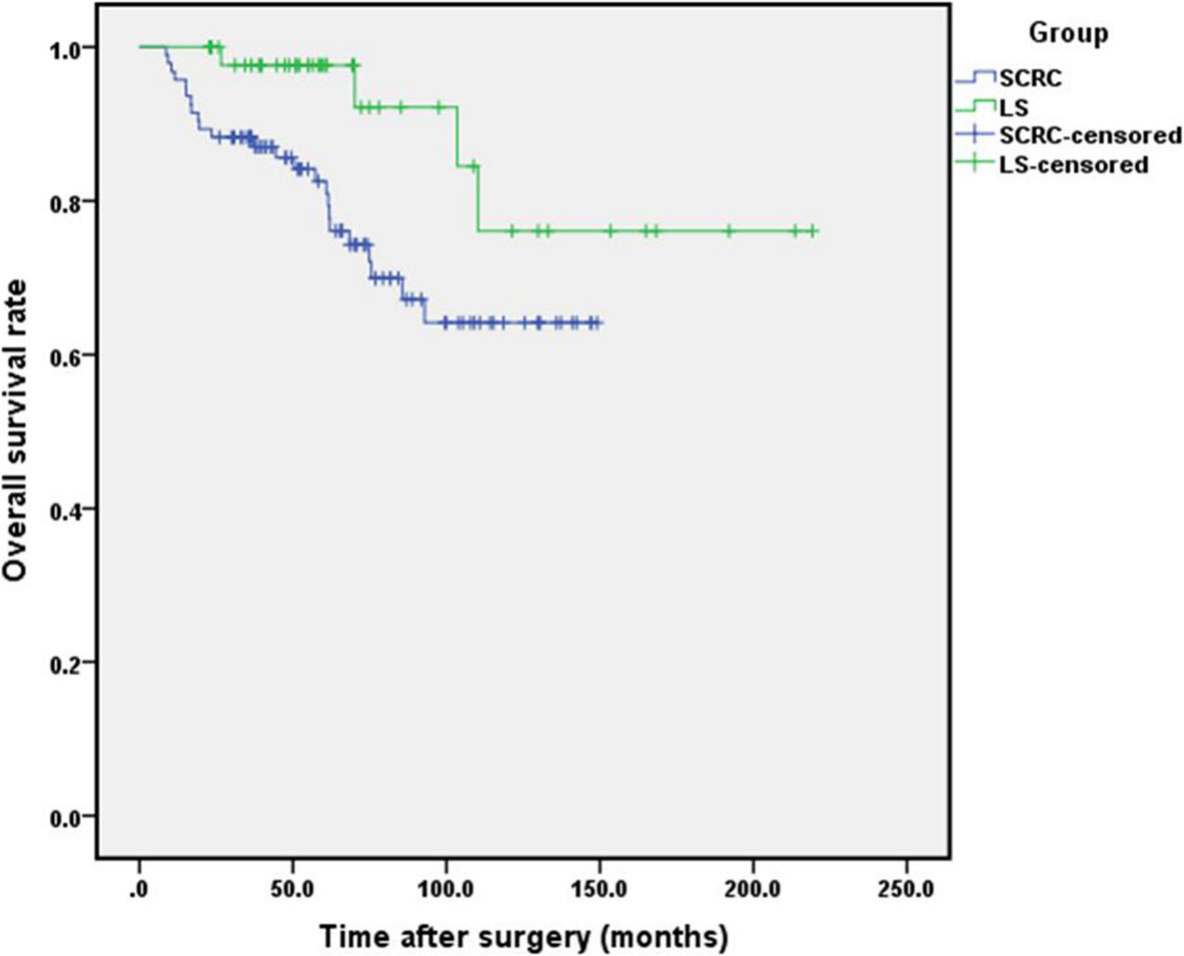
Overall survival curves for patients in LS group and SCRC group. Survivals were evaluated using Kaplan–Meier curves and compared with the log-rank test

The average OS period was (188.4±96.3) months (95% CI: 160.9–216.0) for LS group and (113.8±58.4) months (95% CI: 102.0–126.6) for SCRC group. For LS patients, the 1-, 3-, and 5-year OS rates were 100.0, 97.6, and 97.6%, respectively, which were significantly higher than those of SCRC patients (95.7, 88.3, and 82.6%, respectively; χ^2^ = 4.745; *p* = 0.029) (Fig. 2).

### Prognostic factor analysis for OS and RFS

Univariate analysis showed that sex, etiology, pathological classification, cancerous node, vascular invasion, perineural invasion, TNM stage, and tumor recurrence of primary CRC were significantly associated with OS. Multivariate analysis showed that etiology (LS vs. SCRC), TNM stage, and tumor recurrence primary CRC were independent prognostic factors in OS (Table 3). In addition, univariate analysis showed that pathological classification, differentiation grade, cancerous node, vascular invasion, perineural invasion, and TNM stage were significantly associated with RFS. Multivariate analysis showed that cancerous node, vascular invasion, and TNM stage were the independent prognostic factors in RFS (Table 4).

**Table 3:** Factors associated with overall survival in the patients of LS and SCRC group (univariate and multivariate analysis)

Variable	N. patients	N. events^a^	Univariate analysis		Multivariate analysis	
Total	141	28	χ^2^	*p* value	Risk ratio (95% CI)	*p* value
Sex			4.745	0.028	1.360 (0.525–0.770)	0.527
Male	80	24				
Female	61	4				
Age			0.022	0.883		
< 50	103	21				
≥50	38	7				
CEA			0.482	0.488		
< 5.2	118	21				
≥5.2	23	7				
Etiology			4.745	0.029	0.106(0.025–0.446)	0.002
Lynch syndrome	47	4				
Sporadic CRC	94	24				
Location			3.235	0.357		
Right colon	58	8				
Left colon	61	14				
Rectal	16	3				
Multiple	6	3				
Pathological classification			11.122	0.004	1.504(0.770–2.937)	0.233
Adenocarcinoma	105	21				
Partial mucinous	16	0				
Mucinous adenocarcinoma	20	7				
Differentiation grade			2.611	0.271		
Well	2	0				
Moderately	84	15				
Poorly	55	13				
Cancerous node			13.123	< 0.001	3.285(0.860–12.546)	0.082
Occurrence	9	5				
Absence	132	23				
Vascular invasion			10.163	0.001	3.404(0.915–12.662)	0.068
Occurrence	112	17				
Absence	29	11				
Perineural invasion			41.204	< 0.001	0.707(0.247–2.028)	0.519
Occurrence	21	13				
Absence	120	15				
TNM stage			57.546	< 0.001	2.968(1.478–5.964)	0.002
I	40	2				
II	47	4				
III	46	16				
IV	8	6				
KRAS			0.225	0.636		
Wild type	71	9				
Variant type	70	19				
NRAS			0.011	0.916		
Wild type	136	27				
Variant type	5	1				
Recurrence of primary CRC			106.81	< 0.001	48.917(9.866–242.539)	< 0.001
Occurrence	35	25				
Absence	106	3				

*CEA* Carcinoembryonic antigen, *LS* Lynch syndrome, *SCRC* Sporadic colorectal cancer, *N*. Number, *CI* Confidence interval, *CRC* Colorectal cancer

^a^Events refers to death

**Table 4:** Factors associated with recurrence free survival in the patients of LS and SCRC group (univariate and multivariate analysis)

Variable	N. patients	N. events^a^	Univariate analysis		Multivariate analysis	
Total	141	35	χ^2^	*p* value	Risk ratio (95% CI)	*p* value
Sex			1.173	0.314		
Male	80	21				
Female	61	14				
Age						
< 50	103	26	0.092	0.762		
≥50	38	9				
CEA			0.220	0.639		
< 5.2	118	28				
≥5.2	23	7				
Etiology			1.260	0.262		
Lynch syndrome	47	9				
Sporadic CRC	94	26				
Location			2.018	0.569		
Right colon	58	11				
Left colon	61	17				
Rectal	16	5				
Multiple	6	2				
Pathological classification			19.274	< 0.001	1.345(0.865–2.090)	0.188
Adenocarcinoma	105	24				
Partial mucinous	16	0				
Mucinous adenocarcinoma	20	11				
Differentiation grade			7.260	0.027	0.761(0.343–1.689)	0.502
Well	2	0				
Moderately	84	15				
Poorly	55	20				
Cancerous node			12.595	< 0.001	3.011(1.067–8.498)	0.037
Occurrence	9	6				
Absence	132	29				
Vascular invasion			43.571	< 0.001	0.236(0.109–0.512)	< 0.001
Occurrence	29	21				
Absence	112	14				
Perineural invasion			13.654	< 0.001	1.552(0.679–3.546)	0.297
Occurrence	21	15				
Absence	120	20				
TNM stage			52.055	< 0.001	2.841(1.619–4.986)	< 0.001
I	40	1				
II	47	8				
III	46	19				
IV	8	7				
*KRAS*			0.007	0.934		
Wild type	71	16				
Variant type	70	19				
*NRAS*			0.368	0.544		
Wild type	136	33				
Variant type	5	2				

*CEA* Carcinoembryonic antigen, *LS* Lynch syndrome, *SCRC* Sporadic colorectal cancer, *N*. Number, *CI* Confidence interval, *CRC* Colorectal cancer

^a^Events refers to tumor recurrence of primary CRC

### Subgroup survival

**Fig. 3:**
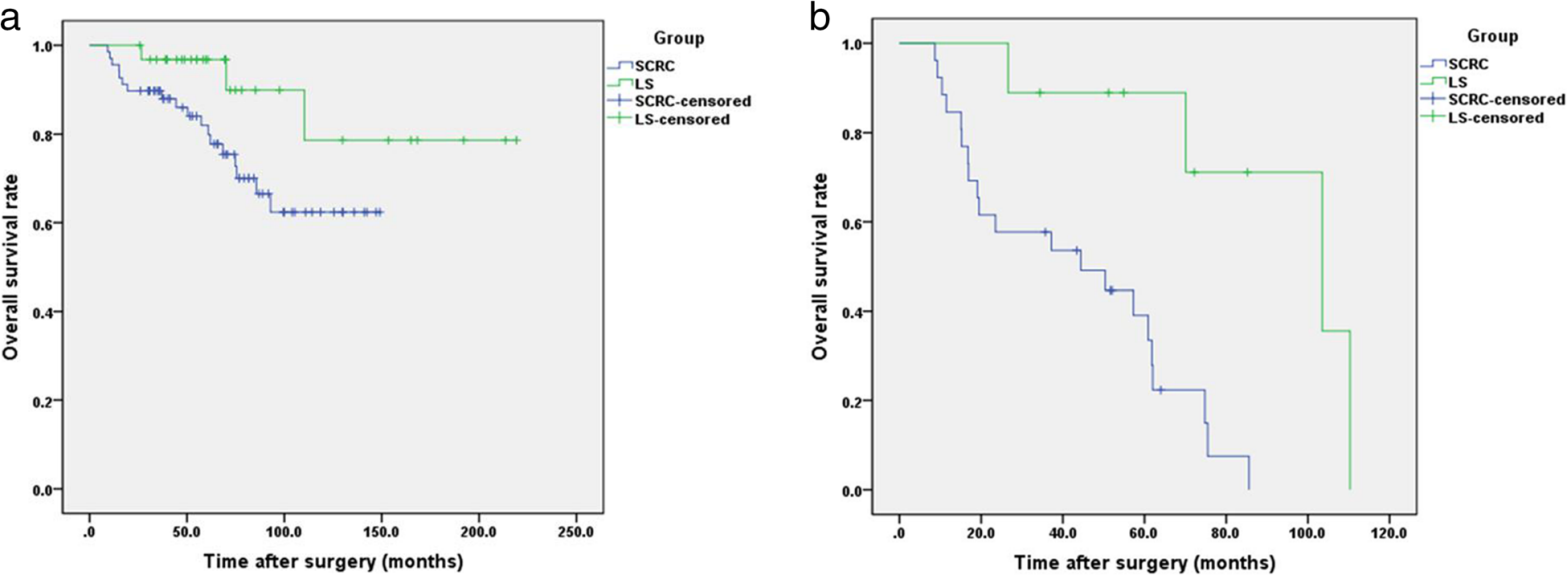
Overall survival curves for patients in subgroup of LS group and SCRC group. Survivals were evaluated using Kaplan–Meier curves and compared with the log-rank test. **a** Early onset CRC patients; **b** CRC patients who developed tumor recurrence

For early-onset (< 50 years) CRC patients, the 1-, 3-, and 5-year OS rates for LS group were 100, 96.8, and 96.8%, respectively, whereas those for the SCRC group were 95.6, 89.7, and 82.0%, respectively. No significant differences were observed between the two subgroups (χ^2^ = 3.332, *p* = 0.068) (Fig. 3a).

For patients who develop tumor recurrence, the 1-, 3-, and 5-year OS rates for LS patients were 100.0, 88.9, and 88.9%, respectively, which were significantly higher than those of SCRC patients (84.6, 57.7, and 39.1%, respectively; χ^2^ = 9.354; *p* = c0.002) (Fig. 3b).

## Discussion

Hereditary background and molecular subtypes are significant factors in the prognosis of CRC patients [24]. As the most common hereditary CRC, LS is characterized by earlier onset, poorly differentiated tumors, and mucinous differentiation [25]. Those clinicopathologic features different from SCRC made it difficult to compare the long-term prognoses between those two subgroups. Using propensity score matching analysis, with the clinical characteristics of LS as baseline and a 1:2 matching ratio between LS and SCRC patients, we achieved comparability between the two groups in the current study. We found that OS period in LS patients was significantly longer than in SCRC patients, which may indicate that long-term prognosis for LS patients is better than that of SCRC patients. We used tumor recurrence of primary CRC to calculate RFS and included metachronous tumor occurrence into the calculations for TFS, because although LS patients have higher metachronous tumor occurrence rates, the occurrence of such second primary tumors is different from primary tumor recurrence in clinical significance. We found that RFS for LS patients is comparable to that for SCRC group, whereas the high proportion of metachronous CRCs and extra-colonic cancers in LS group have remarkably shortened TFS time, resulting in higher TFS rates for the SCRC group.

By analyzing genetic testing, we found that the Chinese population’s LS genotype distribution is similar to distributions reported in western review [6], with *MLH1* and *MSH2* being the majority. However, due to insufficient sample size, no significant differences in the phenotypes and long-term prognosis of each genotype can be found. LS remains underdiagnosed and genetic counseling of LS in the Chinese population is also insufficient. Therefore, prospective studies with larger samples are needed to explore the genotypes and phenotypes of Asian LS patients would also improve screening and follow-up policies for Asian patients.

While comparing primary tumor recurrence rate, previous studies demostrated that LS patients have lower risk of tumor recurrence [26, 27]. However, in the current study, the LS and SCRC groups show no statistical difference in RFS, despite showing a significant tendency of dispersion in their RFS survival curves. A possible reason is that because the majority of tumors in both groups are at early stage. As a result, our follow-up time is not long enough to yield statistical difference in comparison of RFS.

A higher probability of metachronous CRC and extra-colonic tumor is the most prominent feature of LS [6]. In the current study, metachronous CRC and extra-colonic tumors were present in a large portion of LS patients, which significantly shortened the LS group’s TFS. Standardized postoperative monitoring enabled us to detect these metachronous tumors at earlier stages. All patients with second primary tumor occurrence received radical resections and individualized adjuvant treatments, including chemotherapy, targeted therapy, etc., according to each patient’s condition. The longer OS and better prognoses of our LS patients, despite their shorter TFS, may be the result of the above strategy. In fact, research shows that metachronous tumor occurrence should have no significant impact on OS if it can be cured through resection [28]. Our result affirms this phenomenon and highlights the importance of standardized postoperative monitoring for early detection and proper treatment.

The current study confirms what many studies have already proposed, that LS patients have better long-term prognosis than SCRC patients [10–17]. LS-associated tumors may be associated with better prognoses and therapeutic responses, in part because the DNA MMR system, which is missing in CRCs with MSI, is involved in triggering cell death after chemotherapy-induced DNA damage [7]. Several prospective-retrospective analyses demonstrated that adjuvant 5- fluorouracil based chemotherapy was effective in stage II and III CRC displaying MSI [26, 27, 29]. In our study, all patients who developed recurrence received 5-fluorouracil based combined chemotherapy. Subgroup analysis of these patients show that the LS group had longer OS than the SCRC group. This result may indicate that for LS patients who developed recurrence, combined chemotherapy is effective for recurrent LS patients. In recent years, immune checkpoint modulators that directly target the exhaustion-related molecules PD-1/PD-L1 have proven to be effective specifically in tumors displaying the MSI phenotype with an objective response rate of approximately 40% [30]. Thus, immunotherapy or immunotherapy combined with chemotherapy can be another important treatment option in future practice.

During the analysis of prognostic factors in long-term survival, we found that microvascular invasion, cancer nodule occurrence, and the late TNM staging of the first primary CRC were independent risk factors that affect both RFS and OS. These factors have already been widely recognized as adverse prognostic factors [31, 32]. Thus, once an LS patient is identified, the affected relatives should undergo genetic counseling and standardized follow-up monitoring. Diagnosis at earlier stage and timely treatment can mitigate these adverse factors and ensure better survival prognoses.

Lastly, since early-onset CRC is another important characteristic of LS, we conducted a subgroup analysis and comparison of LS and SCRC early-onset CRC patients. However, we found no significant difference in comparison of OS, which indicates that early-onset CRC is an indicator of hereditary CRC in general. Notably, the incidence of early-onset CRC is increasing worldwide [33–35] which indicates that early-onset CRC is a growing concern. Thus, early-onset cancer patients are recommended to undergo genetic testing to screen for LS.

The current study has the following limitations. Firstly, even though propensity score matching was used, selection bias of this retrospective study is still hard to avoid, which may bias the results. Secondly, the LS group contains patients with synchronous primary tumors, a factor which could not be balanced on the baseline and thus may affect the results. Lastly, the sample size for the LS group is relatively small, and would ideally require further accumulation and longer follow-up times.

## Conclusion

The results of the current study indicate that LS patients have better long-term OS than SCRC patients, even though the two groups have comparable RFS. In treating LS, especially for LS patients who developed recurrence of primary CRC, the combined chemotherapy may be the standard treatment. In addition, standardized postoperative monitoring for LS patients enabled us to detect metachronous tumors at earlier stages, which was a guarantee of a favorable prognosis. Lastly, establishing a database for LS patients across Asian populations would allow a deeper understanding of the clinicopathological, molecular-pathological and familial characteristics of LS in Asia, and would provide a stronger theoretical basis for the screening, treatment, and follow-up monitoring of these LS patients and their relatives.

## Data Availability

The datasets used and/or analyzed during the current study are available from the corresponding authors on reasonable request.

## Abbreviations

CRC: Colorectal cancer
CEA: Carcinoembryonic antigen
CI: Confidence interval
IHC: Immunohistochemistry
LS: Lynch syndrome
MMR: Mismatch repair
MSI: Microsatellite instability
OS: Overall survival
RFS: Recurrence free survival
PSM: Propensity score matching
PV: Pathogenic variant
TFS: Tumor free survival
SCRC: Sporadic colorectal cancer
